# Changing the Health Behavior of Patients With Cardiovascular Disease Through an Electronic Health Intervention in Three Different Countries: Cost-Effectiveness Study in the Do Cardiac Health: Advanced New Generation Ecosystem (Do CHANGE) 2 Randomized Controlled Trial

**DOI:** 10.2196/17351

**Published:** 2020-07-28

**Authors:** Jordi Piera-Jiménez, Marjolein Winters, Eva Broers, Damià Valero-Bover, Mirela Habibovic, Jos W M G Widdershoven, Frans Folkvord, Francisco Lupiáñez-Villanueva

**Affiliations:** 1 Open Evidence Research Group Universitat Oberta de Catalunya Barcelona Spain; 2 Department of R&D Badalona Serveis Assistencials Badalona Spain; 3 Smart Homes Eindhoven Netherlands; 4 Department of Medical and Clinical Psychology Tilburg University Tilburg Netherlands; 5 Department of Cardiology Elisabeth-TweeSteden Hospital Tilburg Netherlands; 6 Department of Communication and Cognition Tilburg School of Humanities and Digital Sciences Tilburg University Tilburg Netherlands; 7 Department of Information and Communication Sciences Universitat Oberta de Catalunya Barcelona Spain

**Keywords:** cost-effectiveness, randomized controlled trial, RCT, eHealth, cardiovascular disease, engagement, behavior change, digital health

## Abstract

**Background:**

During the last few decades, preventing the development of cardiovascular disease has become a mainstay for reducing cardiovascular morbidity and mortality. It has been suggested that interventions should focus more on committed approaches of self-care, such as electronic health techniques.

**Objective:**

This study aimed to provide evidence to understand the financial consequences of implementing the “Do Cardiac Health: Advanced New Generation Ecosystem” (Do CHANGE 2) intervention, which was evaluated in a multisite randomized controlled trial to change the health behavior of patients with cardiovascular disease.

**Methods:**

The cost-effectiveness analysis of the Do CHANGE 2 intervention was performed with the Monitoring and Assessment Framework for the European Innovation Partnership on Active and Healthy Ageing tool, based on a Markov model of five health states. The following two types of costs were considered for both study groups: (1) health care costs (ie, costs associated with the time spent by health care professionals on service provision, including consultations, and associated unplanned hospitalizations, etc) and (2) societal costs (ie, costs attributed to the time spent by patients and informal caregivers on care activities).

**Results:**

The Do CHANGE 2 intervention was less costly in Spain (incremental cost was −€2514.90) and more costly in the Netherlands and Taiwan (incremental costs were €1373.59 and €1062.54, respectively). Compared with treatment as usual, the effectiveness of the Do CHANGE 2 program in terms of an increase in quality-adjusted life-year gains was slightly higher in the Netherlands and lower in Spain and Taiwan.

**Conclusions:**

In general, we found that the incremental cost-effectiveness ratio strongly varied depending on the country where the intervention was applied. The Do CHANGE 2 intervention showed a positive cost-effectiveness ratio only when implemented in Spain, indicating that it saved financial costs in relation to the effect of the intervention.

**Trial Registration:**

ClinicalTrials.gov NCT03178305; https://clinicaltrials.gov/ct2/show/NCT03178305

## Introduction

### Background

In the last few decades, prevention at both population and individual levels in patients with established cardiovascular disease (CVD) has become a mainstay for reducing cardiovascular morbidity and mortality [1]. However, CVD remains the leading cause of death globally [2].

One of the cornerstones of CVD prevention is the promotion of lifestyle changes, including physical activity, a healthy diet, and avoidance of unhealthy behaviors such as smoking and drinking alcohol [1]. However, providing patients with relevant information regarding the importance of lifestyle habits seems to be insufficient to prompt these changes and maintain them over time [3]. Instead, it has been suggested that the preventive paradigm should shift from passive to more committed approaches of self-care based on the following three core elements: self-care maintenance, self-care monitoring, and self-care management [4-6].

The emergence of solutions based on information and communication technologies (ICTs), such as telemedicine, has greatly contributed to filling some of the gaps of effective self-care. One of the most classical ICT solutions has been the use of self-monitoring devices in patients with high cardiovascular risk to facilitate successful blood pressure (BP) control [7]. The expansion of mobile apps and their peripheral devices has raised the number of ICT-based interventions aimed at improving not only self-monitoring but also behavior changes in various patient profiles, including older people with high cardiovascular risk [8-10]. The emergence of lifestyle data-driven apps illustrates the increasing interest in this approach in various health care areas [11].

To date, evidence regarding the efficacy of these interventions is still evolving. Clinical guidelines for the prevention of CVD highlight that cost-effectiveness data from randomized controlled trials (RCTs) are scarce, and most data regarding the cost-effectiveness of cardiovascular prevention strategies combine clinical evidence with simulation approaches [1,12,13]. Simulation modeling is currently used to address important issues in clinical practice and health policy that have been very difficult to study within high-quality clinical trials but provide necessary insights for making health care decisions. Nonetheless, assumptions and personal choices are required to conduct simulation modeling, leading to potentially biased outcomes. Transparency in decision-making is therefore critical to adequately understand the observed outcome [14]. In this regard, there is a need for providing the various stakeholders, particularly policy makers, with evidence from nonsimulated research trials to understand the financial consequences of scaling up ICT solutions for health care systems [15]. In this study, we aimed to determine whether the Do Cardiac Health: Advanced New Generation Ecosystem (Do CHANGE) 2 preventive intervention is a cost-effective alternative for patients with CVD in Spain, the Netherlands, and Taiwan.

### The Do CHANGE Project

**Figure 1:**
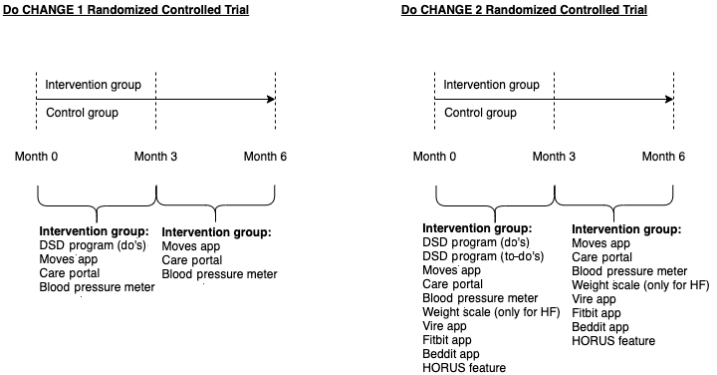
Do CHANGE 1 and 2 randomized controlled trial design including intervention details. Do CHANGE: Do Cardiac Health: Advanced New Generation Ecosystem; DSD: Do Something Different; HF: heart failure.

The Do CHANGE program was developed as an ICT-based alternative for providing health education, which leads to behavioral changes in care recipients [16,17]. The Do CHANGE program consists of a 6-month intervention with a set of devices that include self-monitoring tools and the Do Something Different (DSD) behavior change program (only available during the first 3 months of the intervention), which has been shown to be effective in changing health behaviors in previous studies targeting different populations [18]. The Do CHANGE program included the following two phases: Do CHANGE 1 and Do CHANGE 2, which were assessed in two consecutive RCTs (Figure 1).

Patients included in the Do CHANGE 1 study received the DSD behavior change program, which was provided via text messages on patients’ mobile phones. Behavioral flexibility is associated with a broad range of behavioral repertoires, making people more open to experience and adopt new behaviors [19]. This is achieved by disrupting patients’ daily behavioral routines for a short period (eg, a few seconds) with behavioral prompts (referred to as “do’s”) delivered through patients’ mobile phones. These messages challenge patients to do something different and get out of their comfort zone. Do’s have been developed by a multidisciplinary team including cardiologists and psychologists, ensuring that they apply to the target population and are thus related to daily behaviors or needs. Patients received a total of 32 do’s during the 3-month intervention period (2-3 do’s per week). The program was tailored to the cardiac population with slight differences in the program depending on patients’ primary diagnosis (coronary artery disease, heart failure, and hypertension), as the preferred health behaviors may vary depending on the diagnosis [20]. In order to obtain objective measures on patients’ physical functioning, all participants received a BP monitor, the Moves app (Facebook Inc; to register GPS location), and the Careportal (Docobo Ltd, home monitoring device measuring daily symptoms and an electrocardiogram).

The main additional features of the Do CHANGE 2 compared with the Do CHANGE 1 trial were the greater number of devices for self-monitoring and collecting behavioral information, and the capacity of the DSD program to tailor the behavioral prompts to the actual behavior of the care recipient, thus allowing for a personalized approach. Do CHANGE 2 integrates the principle of theory-driven behavioral change techniques, which can guide behavior change, within the offered interventions [21]. As a natural evolution from the Do CHANGE 1 trial, the second phase aimed to increase the ability of a person to express behavior in a more context-dependent way [22], thus being more open to experience and increasing the likelihood of adopting new behaviors [19].

Care recipients perceived the Do CHANGE 1 program as helpful and easy to use; however, it failed to prompt relevant lifestyle changes (measured with the Health Promotion Lifestyle Profile-II questionnaire) compared with treatment as usual (TAU) [20]. The Do CHANGE 2, based on a more personalized approach, resulted in a relevant change in lifestyle behavior over time in the intervention group. In addition, the intervention was perceived as useful and feasible by patients and health care professionals [23]. In order to provide a broader perspective of the effects of this program, we present herein the results of the cost-effectiveness analysis of the Do CHANGE 2 compared with TAU.

## Methods

### Trial Design and Patients

This was a multisite RCT to assess the cost-effectiveness of an ICT-based program to change behavior in patients with CVD compared with TAU. Local clinical specialists and research assistants recruited adult patients treated in the following three hospitals in three different countries: *Badalona Serveis Assistencials* (Spain), *Elisabeth TweeSteden Ziekenhuis* (the Netherlands), and *Buddhist Tzu-Chi Dalin General Hospital* (Taiwan). The planned sample size based on the available project resources was 75 patients for Spain, 75 for the Netherlands, and 100 for Taiwan. Once accepted to participate in the study, patients at each study site were randomized to receive either the TAU or the Do CHANGE 2 intervention. The primary outcomes were lifestyle change and quality of life. Additionally, behavioral flexibility was considered a mediator variable in this relationship. As the project aimed to provide proof of concept and examine the feasibility of the intervention, no sample size calculation was performed a priori. Recruiting a comparable number of patients across the countries was considered relevant to provide proof of concept. The details regarding the study patients and trial design are described in the report by Habivovic et al [16].

The most remarkable changes from the original study protocol (Do CHANGE 1) were the changes in the DSD program (moving from predefined messages according to the patient psychological profile to nudges tailored according to their behavior as gathered by the measurement devices), the addition of two new wearable devices (Beddit [Apple] and Fitbit [Fitbit Inc]), and the Vire app (Do CHANGE app). Considering the importance of weight in heart failure (HF), patients with this diagnosis also received a weight scale.

#### Inclusion Criteria

Participants were screened from adult patients (aged 18-75 years) who had been primarily diagnosed with either hypertension (ie, systolic BP [SBP]/diastolic BP [DBP] ≥140/90 mmHg in two consecutive measurements), coronary artery disease (ie, occurrence of myocardial infarction or angina pectoris, or previous percutaneous coronary intervention and/or coronary artery bypass graft surgery), or symptomatic HF (ie, New York Heart Association class I-IV). Patients also had to have two or more of the following risk factors: increased cholesterol, smoking, diabetes, sedentary lifestyle, and psychosocial risk factors. The presence or absence of each of the risk factors was assessed following the local guidelines in each participant country. For HF patients, additional inclusion criteria were a diagnosis of systolic or diastolic HF and the presence of HF symptoms (eg, exhaustion, shortness of breath, and chest pain). Other general inclusion criteria were an adequate level of the native language, access to the internet at home, having a smartphone compatible with the apps used in the study, and having the skills necessary to use a personal computer and a smartphone.

#### Exclusion Criteria

Patients with life expectancy less than 1 year, life-threatening comorbidities, a history of psychiatric diseases other than anxiety and depression, and relevant cognitive impairments and those on the waiting list for heart transplantation were excluded from the study.

The reasoning for establishing the exclusion criteria was to prevent the inclusion of patients whose disease severity may critically increase during the intervention. These patients may perceive participation as an extra burden, are more likely to drop out due to illness-related complaints or early mortality, and may be less likely to benefit from a lifestyle intervention owing to severe comorbidities. Patients with mental illness were also excluded because the intervention might become stressful and trigger symptoms in these patients. The selected exclusion criteria are in line with the DO CHANGE 1 trial, safeguarding that the study is not perceived as a burden and meets patients’ needs as much as possible.

### Intervention

The Do CHANGE 2 program implemented in this trial was similar to that described by Broers et al for Do CHANGE 1 [20]. Patients randomized to the intervention group received devices for measuring key clinical parameters needed for monitoring their CVD, such as a BP monitor, weight scale (in HF patients only), and the Careportal, which allowed monitoring of daily symptoms and an electrocardiogram. The patient’s location was monitored by the Moves app. In addition to the aforementioned ICT solutions (also used in the Do CHANGE 1 program), the Do CHANGE 2 program included the Vire app, a purpose-designed app to integrate the input from all the monitoring devices, so that the patient could interact with a unique easy-to-use source of information. The app integrates the information coming from the following apps: the Beddit app (provided with the device under the mattress cover sheet) aimed at monitoring sleep efficiency, the Fitbit app (with the wristband) aimed at measuring physical activity through step count, and the HORUS feature embedded in and aimed at collecting pictures of the different meals of the patients in order to provide diet recommendations. Study participants in the Do CHANGE 2 intervention group were also provided with leaflets (Multimedia Appendix 1) and multimedia resources explaining the use of the Do CHANGE environment.

Like in the Do CHANGE 1 program, patients in the intervention group received a 3-month behavior change program. The program was based on providing care recipients with short messages aimed at disturbing daily routines. Messages were delivered through their mobile phones and suggested them to “do something different.” However, unlike the Do CHANGE 1 program, in the Do CHANGE 2 program, behavioral nudges were not only predefined according to the patient’s personality profile but also tailored to the patient’s behavior, as recorded by the monitoring devices. These behavior-driven messages called to-do’s were delivered to the patients based on their current functioning. Patients receive their do’s and to-do’s through the Careportal or the Vire app, or via SMS, depending on patients’ preferences [16].

The GPS data from the Moves app and activity data from the Fitbit device were used to calculate higher abstraction scores called *activity*, *variety*, and *social opportunities*. The to-do’s were tailored based on the trends of these scores over time (eg, a patient with declining activity would receive a message that focuses on increasing activity). The granularity of this system is not restricted to one score; alternatively, new scores are calculated for each update of data, and the system determines whether the score is a “target” for a message. Multiple combinations of targets are possible; therefore, a to-do can tackle both *variety* and *social opportunities* if needed based on the scores. A detailed description of the construction process of to-do’s can be found in previously published work [23,24].

Besides receiving personalized prompts (eg, based on activity levels), patients were contacted each week by the research assistant to check how everything was going and to provide dietary coaching. This might have greatly contributed to the high adherence rate during the first 3 months, as they received personalized feedback. After this period, patients were not contacted in person anymore; however, they were allowed to keep all the devices (eg, Fitbit, Beddit, etc) in order to monitor their behavior for the remaining 3 months.

### Cost-Effectiveness Analysis

**Figure 2:**
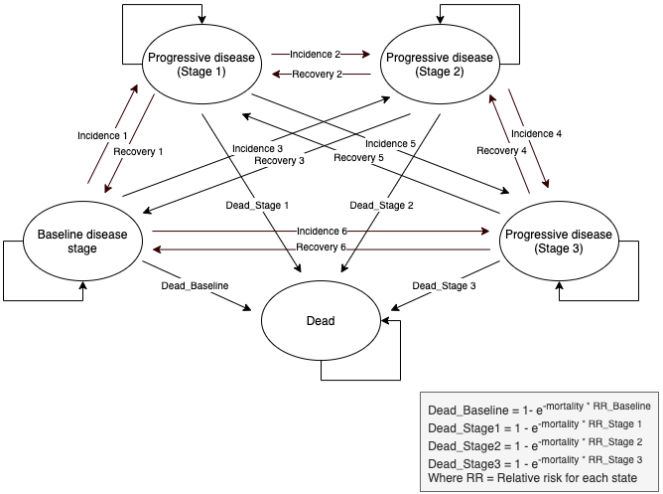
Markov model of five health states applied for the Do CHANGE cost-effectiveness analysis. Do CHANGE: Do Cardiac Health: Advanced New Generation Ecosystem.

The cost-effectiveness analysis of the Do CHANGE 2 RCT was conducted using the Monitoring and Assessment Framework for the European Innovation Partnership on Active and Healthy Ageing (MAFEIP) tool [25]. The MAFEIP tool performs a cost-utility analysis through a web app that analyzes incremental costs and effects. The cost-effectiveness estimates are based on the principles of decision analytic modeling and Markov models that assess the impacts that health-related innovations have in terms of health outcomes and resource usage. For Do CHANGE 2, we parametrized the tool on a Markov model of five health states from the perspective of the three service providers (Figure 2) [26].

The MAFEIP tool requires the user to provide the following three main types of inputs: (1) the health states and the corresponding transition probabilities between them, (2) the costs, and (3) the utility, for which the EuroQol five-dimension three-level (EQ-5D-3L) questionnaire was used as recommended by the National Institute for Health and Care Excellence [27].

In order to estimate the incremental health gain from a particular intervention delivered, the defined model needs to be run twice. Once with parameter estimates for the respective intervention under assessment (ie, Do CHANGE 2), and once with parameters corresponding to the standard care scenario (ie, TAU). In the model, these two scenarios may differ in terms of transition probabilities (disease incidence, recovery, and mortality), as well as the utility weight and health care and societal costs related to the health states. When the model simulates a hypothetical cohort of patients moving between these health states over time, the differences in survival, utility, and cost accumulate until reaching an estimate of the incremental costs (ΔC) and health effects (ΔE) that can be expected from the intervention under evaluation. Therefore, the tool can be used to estimate the incremental cost-effectiveness ratio (ICER=ΔC/ΔE) or the incremental net monetary benefit (ΔE×λ−ΔC) of one intervention compared with another, where λ is defined as the willingness-to-pay (WTP) threshold for an additional unit of health gain.

Besides the transition probabilities among health states, the utility, and the cost, the tool allows the user to include the relative risks for mortality, the discount rates, and the time horizon for the analysis (cycle length). The parameters included in this analysis and their assessments during the study are explained in detail below.

#### Definition of Health States

The first stage in the construction of a Markov model is defining the different states of the disease in relation to the important clinical and economical effects of the disease. Evidence suggests that high BP is the predominant risk factor for CVD [28]. Following the scientific evidence and for the purpose of the Do CHANGE assessment, the health states were established based on SBP and DBP, according to the classification of the American Heart Association [29] as follows: baseline disease stage (SBP <120 mmHg and DBP <80 mmHg), progressive disease stage 1 (SBP 120-129 mmHg and DBP <80 mmHg), progressive disease stage 2 (SBP 130-139 mmHg or DBP 80-89 mmHg), progressive disease stage 3 (SBP ≥140 mmHg or DBP ≥90 mmHg), and death. On a side note, the latest stage was not included, as it is considered to be a hypertensive crisis (SBP >180). The transition probabilities were calculated based on the changes between the initial health states (at baseline) and those at 3 months. These transitions can be of incidence (ie, the annual probability for an individual to move from baseline to each progressive stage of the disease) and recovery (ie, the annual probability of improving).

#### Cost Estimate

The following two types of costs were considered for both study groups: (1) health care costs (ie, costs associated with the time spent by health care professionals on service provision, including consultations, unplanned hospitalizations, etc) and (2) societal costs (ie, costs associated with the time spent by patients and informal caregivers on care activities).

The data collected by the research team in each country were provided in local currency units (Euro for both Spain and the Netherlands and New Taiwan Dollars for Taiwan). Taiwan prices were converted into a common basis of 2018 Euros using simple exchange rate conversion factors, reflecting the average market exchange rate between New Taiwan Dollars and Euros during the year in question (NT $1=€0.02862). A currency exchange rate of €1=US $1.12 is applicable (average exchange rate for 2018).

For computing the time spent by health care professionals, we considered an average duration of 15 minutes and 25 minutes for a visit to a general practitioner and specialist, respectively. The personnel cost was established based on the average cost for one full‐time employee, including employer contributions to social security. The average hourly costs were €29.23 (Spain), €59 (the Netherlands), and €16.88 (Taiwan) for a general practitioner; €20.79 (Spain), €40 (the Netherlands), and €6.33 (Taiwan) for a nurse; and €34.81 (Spain), €113.50 (the Netherlands), and €84.91 (Taiwan) for a specialist. The estimations of cost per bed‐day for hospitalizations were €733.56 (Spain) and €1853.57 (the Netherlands), which were obtained by dividing the expenditure for inpatient curative care in hospitals by hospital bed‐days for services of curative care (both publicly available) [30]. The corresponding costs for Taiwan were calculated by dividing the average expenses of hospitalization by the number of hospital days, which was €342.50 [31].

Societal costs differed according to the study group. For patients allocated to the control group, we considered the extra travel time spent by patients and caregivers in usual care compared with Do CHANGE 2, whereas for those allocated to the intervention group, we considered the time spent by patients using the service.

Additionally, for patients in the Do CHANGE group, the following costs were added: time spent by professionals in service development and training (4 hours per professional, divided by the number of randomized subjects), time spent by nurses in training patients (30 minutes per participant) and installing the Do CHANGE service ecosystem (45 minutes per patient), and the cost of the devices (including taxes). Data are provided in Euro (2018).

#### Utility Calculation

Utility was estimated using the EQ-5D-3L tool [32]. The EQ-5D is a standardized questionnaire-based measure of self-rated health-related quality of life developed by the EuroQol Group to provide a simple and generic measure widely used for both clinical and economic appraisals. In the case of the Do CHANGE 2 project, we used the EQ-5D-3L version, which was administered at baseline and at 3 and 6 months.

The resulting scores of the questionnaire were weighted using the trade-off method previously described for Spain [33], the Netherlands [34], and Taiwan [35]. The EQ-5D health states, defined by the EQ-5D descriptive system, were subsequently converted into a single summary index by applying specific weights to each of the levels in each dimension of quality of life. The index was calculated by deducting the appropriate weights from 1, which was the value assigned to full health. In the case of the cost-effectiveness analysis of the Do CHANGE intervention, our interest was to measure the change over time, rather than the absolute values. Therefore, we calculated the changes in utility for each of the five health states and for each of the study conditions and added a common initial measure for the whole sample to each of them. The MAFEIP requires EQ-5D utility scores combined with time indicators to compute quality-adjusted life-years (QALYs) automatically.

#### Relative Risks of Mortality, Discount Rates, and Time Horizon

The MAFEIP tool allows mortality rates to be internally calculated by using the all-cause mortality rates (age- and sex-dependent) extracted from the Human Mortality Database. The relative risk of mortality is a measure that estimates the mortality in a specific population (eg, people who participated in the Do CHANGE 2 study) compared with (ie, divided by) the mortality in a reference population or condition (in this case, from the Human Mortality Database). The reference condition considers CVD mortality for the population of the specified country (ie, Spain, the Netherlands, and Taiwan).

The discount factors for costs and effects are used to estimate outcomes while taking into account the future costs and health effects, that is, adjusting for differences in the timing of costs (expenditure) compared with health benefits (outcomes). Therefore, adequately applied discount factors express future costs or benefits at today’s equivalent value. In Do CHANGE 2, we followed the recommendations from the Health Technology Assessment authorities in each country [36-40]. The discount factors for costs and health outcomes applied in Do CHANGE 2 were 3% for both costs and health outcomes in Taiwan and Spain, and 4% for costs and 1.5% for health factors in the Netherlands.

Finally, the MAFEIP framework allows specifying the number of cycles that the model will run, which represents the timeframe in which the impact of the intervention will be evaluated. Markov models are used to simulate both short-term and long-term processes (ie, CVDs) [41]. In the case of Do CHANGE 2, we wanted to see estimates of the incremental costs (and effects) of the intervention in a time horizon of 5 years. The cycle length we selected is not in line with CVD’s etiology (ie, a long disease development process) [41] but considers the nature of the intervention and the maximum time frame it can be sustained in light of the deprecation of the wearable and medical device technology that was used.

#### WTP Threshold

The balance between the economic benefit and clinical effectiveness varies and is entirely dependent on the relationship between the ICER and the threshold value the society is willing to pay at a specific point of time, which is known as the WTP threshold. The fact that WTP thresholds can be specified after the ICER is calculated raises concerns about researchers selecting WTP thresholds that suit their hypothesis, hence compensating for technology of relatively lower value [42].

While there is an agreement about CVDs being preventable to a certain extent, there has also been a discussion as to whether prevention interventions offer good value for money. Previous research has shown a positive relation between lower lifetime risk for CVD mortality and increased survival and quality of life [43]. Prevention strategies can bring relevant benefits at lower costs relative to most treatment options provided that their cost-effectiveness value is almost always below the accepted societal WTP [44].

For the Do CHANGE 2, we selected a WTP threshold of €15,000/QALY for the three countries, not corresponding to the value recommended by local Health Technology Assessment guidelines. The WTP threshold is lower in all cases. We set a lower WTP threshold in order to avoid the concerns mentioned above and to fit the results of the technology, and considering comparisons with other preventive interventions.

### Data Collection and Analysis

The questionnaires, as defined in the study protocol, were loaded into the web tool LimeSurvey [45] and collected by local research assistants. Data from the medical devices (built-in electrocardiogram monitor, blood pressure meter, and weight scale) were collected through the Careportal. The data generated by the wearable devices (Fitbit and Beddit) were continuously monitored and integrated through the Vire app. Information regarding resource consumption (eg, hospitalization costs) was collected by local research assistants from the local electronic medical records.

IBM SPSS Statistics for Windows Version 21.0 (IBM Corp) was used to perform the statistical analyses of the effectiveness study, and R (R Core Team 2018) and RStudio (RStudio Team 2016) were used to calculate the transition probabilities and utilities. We used the MAFEIP tool to perform the cost-effectiveness analysis.

## Results

### Study Participants

**Figure 3:**
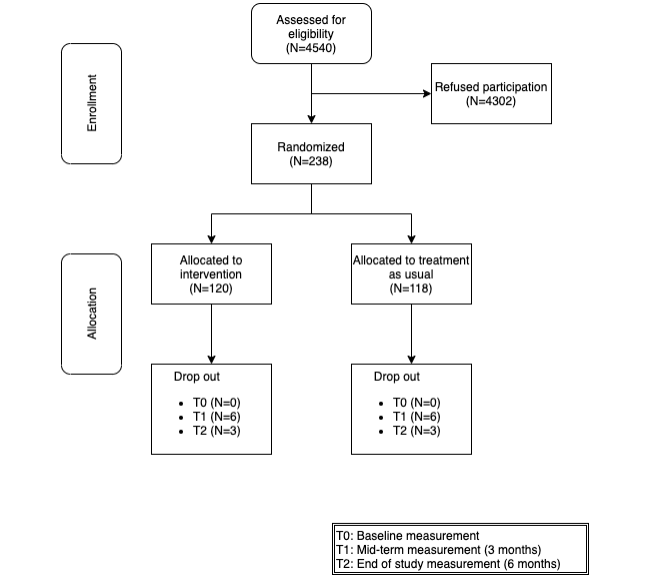
Flow chart of participant recruitment (aggregated numbers for Spain, the Netherlands, and Taiwan).

Figure 3 shows the overall flow chart of participant recruitment in the Do CHANGE 2 project. Of the 4540 patients assessed for eligibility at all three sites, 238 were enrolled in the study (120 in the intervention group [Do CHANGE] and 118 in the control group).

Owing to relevant differences in patient recruitment strategies between sites, most patients screened in Spain and the Netherlands agreed to participate, whereas many patients in Taiwan refused to participate. Based on retrospective investigation, it appeared that all inclusion criteria in Taiwan were checked after the patients were approached for participation. Hence, the number of patients that were approached appeared to be much higher. In Spain and the Netherlands, patients who fulfilled the basic inclusion criteria were offered to participate. If the same strategy was applied in Taiwan, the refusal rate would have been much lower. Both Spain and the Netherlands met the target number of participants as defined in the project plan, whereas Taiwan did not reach the planned number of participants.

Eighteen patients dropped out before the end of the 6-month follow-up, demonstrating a very high adherence rate. Owing to the personalized nature of the intervention (eg, relevant behavioral prompts and personalized feedback), we expected the adherence to be high. We believe that the nature of the 3-month intervention, where blended and personalized care was provided, combined with the monitoring devices after that period contributed to the high adherence. Patients were engaged in their health management, and therefore, they might be more willing to proceed with monitoring.

Of the 114 participants who were in the program for at least 3 months, 72 (60%) claimed to have carried out all the nudges provided by the DSD and 86 (72%) reckoned the program was useful. Reasons for not adhering to the program were mainly having no time (8/84, 6.7%), not feeling like it (2/84, 1.7%), and falling ill (2/84, 1.7%). One of the participants who quit the program disclosed that being confronted with the illness on a daily basis became too stressful. Moreover, in some cases, the confrontation for some partners to deal with the illness of their husband or wife caused anxiety.

Table 1 summarizes the main demographic characteristics of the three participating countries. None of the variables collected showed relevant systematic differences between study conditions at baseline, except for Spain, with participants in the Do CHANGE 2 group being younger (Do CHANGE 2 group vs TAU group: mean 53.8 years, SD 15.8 years vs mean 67.4 years, SD 7.5 years), having a higher education (mean 14.5 years, SD 6.3 years vs mean 9.1 years, SD 5.5 years), and showing a higher employed proportion (17/37, 45.9% vs 4/37, 10.8%). Multimedia Appendix 2 presents the clinical characteristics of the study sample, medication, and psychological symptoms. The only significant difference at baseline was observed in psychotropic medication for participants in the TAU group in Spain (*P*=.03), which is consistent with the population in the control group being older.

**Table 1:** Demographic baseline characteristics of the total sample (N=238).

Characteristic		Spain (N=75)	The Netherlands (N=75)	Taiwan (N=88)	Total (N=238)
**Sample size, n (%)**					
	Do CHANGE^a^ 2	38 (50.7)	38 (50.7)	44 (50.0)	120 (50.4)
	TAU^b^	37 (49.3)	37 (49.3)	44 (50.0)	118 (49.6)
	Total	75 (100.0)	75 (100.0)	88 (100.0)	238 (100.0)
**Age (years), mean (SD)**					
	Do CHANGE 2	53.8 (15.8)	63.0 (9.2)	58.2 (9.9)	58.3 (12.3)
	TAU	67.4 (7.5)	63.9 (7.4)	56.7 (9.1)	62.3 (9.2)
	Total	60.5 (14.1)	63.4 (8.3)	57.5 (9.5)	60.3 (11.1)
**Gender (male), n (%)**					
	Do CHANGE 2	27 (71.1)	32 (84.2)	30 (68.2)	89 (74.2)
	TAU	19 (51.4)	29 (78.4)	38 (86.4)	86 (72.9)
	Total	46 (61.3)	61 (81.3)	68 (77.3)	175 (73.5)
**Education (years), mean (SD)**					
	Do CHANGE 2	14.5 (6.3)	12.9 (5.1)	14.9 (5.5)	14.1 (5.7)
	TAU	9.1 (5.5)	13.16 (7.9)	16.4 (5.0)	13.1 (6.9)
	Total	11.8 (6.5)	13.0 (6.6)	15.7 (5.3)	13.6 (6.3)
**Marital status (partner), n (%)**					
	Do CHANGE 2	27 (71.1)	34 (89.5)	39 (88.6)	100 (83.3)
	TAU	27 (73.0)	33 (89.2)	42 (95.5)	102 (86.4)
	Total	54 (72.0)	67 (89.3)	81 (92.0)	202 (84.9)
**Working status (paid job), n (%)**					
	Do CHANGE 2	17 (45.9)	13 (34.2)	26 (59.1)	56 (46.7)
	TAU	4 (10.8)	16 (43.2)	28 (63.6)	48 (40.7)
	Total	21 (28.0)	29 (38.7)	54 (61.4)	104 (43.7)
**Smoking (yes), n (%)**					
	Do CHANGE 2	7 (18.4)	3 (7.9)	2 (4.5)	12 (10.0)
	TAU	5 (13.5)	7 (18.9)	4 (9.1)	16 (13.6)
	Total	12 (16.0)	10 (13.3)	6 (6.8)	28 (11.8)

^a^Do CHANGE: Do Cardiac Health: Advanced New Generation Ecosystem.

^b^TAU: treatment as usual.

### Model Input

The model input for the MAFEIP tool included data regarding the health states (and transition probabilities) of study participants, the costs associated with each study group, and the utility estimate. Table 2 summarizes the distribution of study participants across the MAFEIP health states at study start, as well as the transition probabilities between these states, computed by considering data recorded at month 3. Table 3 summarizes the total health care and societal costs for each group and each state. The detailed amounts for each type of health care and societal cost are provided in Multimedia Appendix 3. The specific costs associated with the implementation of the Do CHANGE environment are presented in Table 4. The utility values calculated from the EQ-5D-3L scores and the estimated utility computed by adding the initial common measure are described in Table 5. No systematic differences were observed between study conditions at baseline. The utility values for the whole study sample in Spain, the Netherlands, and Taiwan were 0.897, 0.842, and 0.854, respectively.

**Table 2:** Frequency and percentage of patients across the various health states (N=207).

Variable		Do CHANGE 2^a,b^ (N=92)						TAU^b,c^ (N=115)					
		Spain (n=27)		The Netherlands (n=29)		Taiwan (n=36)		Spain (n=36)		The Netherlands (n=37)		Taiwan (n=42)	
**Health states at study start, n (%)^d^**													
	Baseline disease stage		5 (19.4%)		4 (14.3%)		9 (25.0%)		5 (13.5%)		5 (13.3%)		8 (18.6%)
	Progressive disease stage 1		3 (11.1%)		1 (2.9%)		0		1 (2.7%)		2 (6.7%)		3 (7.0%)
	Progressive disease stage 2		10 (36.1%)		9 (31.4%)		15 (40.9%)		17 (46.0%)		10 (26.7%)		19 (44.2%)
	Progressive disease stage 3		9 (33.3%)		15 (51.4%)		12 (34.1%)		14 (37.8%)		20 (53.3%)		13 (30.2%)
**Transition probabilities, %**													
	Incidence 1 (baseline disease stage to progressive disease stage 1)		14.3%		0.0%		18.2%		80.0%		25.0%		12.5%
	Recovery 1 (progressive disease stage 1 to baseline)		0.0%		0.0%		0.0%		100.0%		0.0%		0.0%
	Incidence 2 (baseline disease stage to progressive disease stage 2)		14.3%		20.0%		0.0%		20.0%		0.0%		37.5%
	Recovery 2 (progressive disease stage 2 to baseline)		7.7%		0.0%		5.6%		17.7%		0.0%		10.5%
	Incidence 3 (progressive disease stage 1 to stage 2)		100.0%		0.0%		0.0%		0.0%		0.0%		100.0%
	Recovery 3 (progressive disease stage 2 to stage 1)		38.5%		0.0%		11.1%		29.4%		12.5%		10.5%
	Incidence 4 (baseline disease stage to progressive disease stage 3)		14.3%		0.0%		0.0%		0.0%		50.0%		12.5%
	Recovery 4 (progressive disease stage 3 to baseline)		0.0%		0.0%		0.0%		0.0%		0.0%		0.0%
	Incidence 5 (progressive disease stage 1 to stage 3)		0.0%		0.0%		0.0%		0.0%		0.0%		0.0%
	Recovery 5 (progressive disease stage 3 to stage 1)		0.0%		0.0%		0.0%		0.0%		0.0%		7.7%
	Incidence 6 (progressive disease stage 2 to stage 3)		38.5%		27.3%		16.7%		5.9%		0.0%		47.4%
	Recovery 6 (progressive disease stage 3 to stage 2)		50.0%		5.6%		33.3%		42.9%		0.0%		30.8%

^a^Do CHANGE: Do Cardiac Health: Advanced New Generation Ecosystem.

^b^Distribution of study participants at study start and the corresponding transition probabilities (in percentage).

^c^TAU: treatment as usual.

^d^Baseline disease stage: systolic blood pressure (SBP) <120 mmHg and diastolic blood pressure (DBP) <80 mmHg; Progressive disease stage 1: SBP 120-129 mmHg and DBP <80 mmHg; Progressive disease stage 2: SBP 130-139 mmHg or DBP 80-89 mmHg; Progressive disease stage 3: SBP ≥140 mmHg or DBP ≥90 mmHg.

**Table 3:** Total health care and societal costs for each of the study groups (N=207).

Variable		Do CHANGE 2^a,b^ (N=92)				TAU^b,c^ (N=115)				
		Spain (n=27)	The Netherlands (n=29)	Taiwan (n=36)		Spain (n=36)	The Netherlands (n=37)		Taiwan (n=42)	
**Health care costs^d^**										
	Baseline disease stage	299.90	489.82	156.94	646.08			343.97		114.25
	Progressive disease stage 1	729.25	166.43	244.96	1284.41			88.76		71.45
	Progressive disease stage 2	942.39	240.36	161.58	2381.76			313.00		93.44
	Progressive disease stage 3	2176.32	240.36	138.67	3484.41			88.76		114.94
**Societal costs^d^**										
	Baseline disease stage	309.61	512.90	158.94	648.48			367.43		113.35
	Progressive disease stage 1	737.38	198.51	247.90	1289.75			76.53		70.71
	Progressive disease stage 2	953.98	277.55	163.44	2386.12			323.81		93.06
	Progressive disease stage 3	2198.96	287.20	140.62	3485.76			73.47		115.09

^a^Do CHANGE: Do Cardiac Health: Advanced New Generation Ecosystem.

^b^Data are presented in € (2018; €1=US $1.12). The detailed costs of each category are provided in Multimedia Appendix 3.

^c^TAU: treatment as usual.

^d^Baseline disease stage: systolic blood pressure (SBP) <120 mmHg and diastolic blood pressure (DBP) <80 mmHg; Progressive disease stage 1: SBP 120-129 mmHg and DBP <80 mmHg; Progressive disease stage 2: SBP 130-139 mmHg or DBP 80-89 mmHg; Progressive disease stage 3: SBP ≥140 mmHg or DBP ≥90 mmHg.

**Table 4:** Costs associated with the implementation of the Do CHANGE 2 intervention (N=92).

Variable	Spain^a^ (n=27)	The Netherlands^a^ (n=29)	Taiwan^a^ (n=36)
Time spent by professionals^b^ (overhead of 18%)	50.02	99.79	19.19
Time spent by specialists (service development, receiving training, and adaptation)	1.86	7.12	4.53
Time spent by nurses (service development, receiving training, and adaptation)	1.39	2.67	0.42
Time spent by nurses on training provision to patients	25.99	50	7.91
Time spent by nurses on installation of the Do CHANGE^c^ ecosystem	20.79	40	6.33
Cost of the set of devices included within the Do CHANGE ecosystem	748.99	748.99	748.99
Total	799.01	848.78	768.18

^a^Data are presented in € (2018; €1=US $1.12).

^b^For the personnel cost, we use the average cost for one full‐time employee including employer contributions to social security. The average hourly costs are as follows: €29.23 (Spain), €59 (the Netherlands), and €16.88 (Taiwan) for a physician; €20.79 (Spain), €40 (the Netherlands), and €6.33 (Taiwan) for a nurse; and €34.81 (Spain), €113.50 (the Netherlands), and €84.91 (Taiwan) for a specialist.

^c^Do CHANGE: Do Cardiac Health: Advanced New Generation Ecosystem.

**Table 5:** Calculation of utility (N=207).

Disease stage^a^ and assessment		Spain (N=63)						The Netherlands (N=66)						Taiwan (N=78)						
		Do CHANGE^b^ 2 (N=27)		TAU^c^ (N=36)		*P* value^d^		Do CHANGE 2 (N=29)		TAU (N=37)		*P* value^d^			Do CHANGE 2 (N=36)		TAU (N=42)		*P* value^d^	
**Baseline disease stage**																				
	M0^e^		0.896		0.869		.74		0.854		0.936		.40			0.875		0.638		.01
	M3^f^		0.900		0.950		.49		0.931		0.904		.83			0.911		0.847		.59
	Δ^g^		0.004		0.081				0.077		−0.032					0.036		0.209		
**Progressive disease stage 1**																				
	M0		0.871		0.719		—^h^		0.861		0.807		—			0.726		1		—
	M3		0.853		0.898		.57		0.861		0.904		—			0.726		1		.19
	Δ		−0.018		0.179				0		0.097					0		0		
**Progressive disease stage 2**																				
	M0		0.912		0.875		.49		0.896		0.821		.23			0.877		0.895		.75
	M3		0.938		0.853		.07		0.886		0.825		.42			0.841		0.883		.56
	Δ		0.026		−0.022				−0.010		0.004					−0.036		−0.012		
**Progressive disease stage 3**																				
	M0		0.964		0.889		.09		0.805		0.843		.42			0.832		0.870		.62
	M3		0.944		0.866		.21		0.852		0.872		.64			0.766		0.838		.32
	Δ		−0.020		−0.023				0.047		0.029					−0.066		−0.032		

^a^Baseline disease stage: systolic blood pressure (SBP) <120 mmHg and diastolic blood pressure (DBP) <80 mmHg; Progressive disease stage 1: SBP 120-129 mmHg and DBP <80 mmHg; Progressive disease stage 2: SBP 130-139 mmHg or DBP 80-89 mmHg; Progressive disease stage 3: SBP ≥140 mmHg or DBP ≥90 mmHg.

^b^Do CHANGE: Do Cardiac Health: Advanced New Generation Ecosystem.

^c^TAU: treatment as usual.

^d^A *P* value <.05 is considered significant.

^e^M0: baseline assessment.

^f^M3: assessment at 3 months.

^g^Δ: M3 – M0.

^h^Not enough data to calculate a *P* value*.*

### Cost-Effectiveness

**Figure 4:**
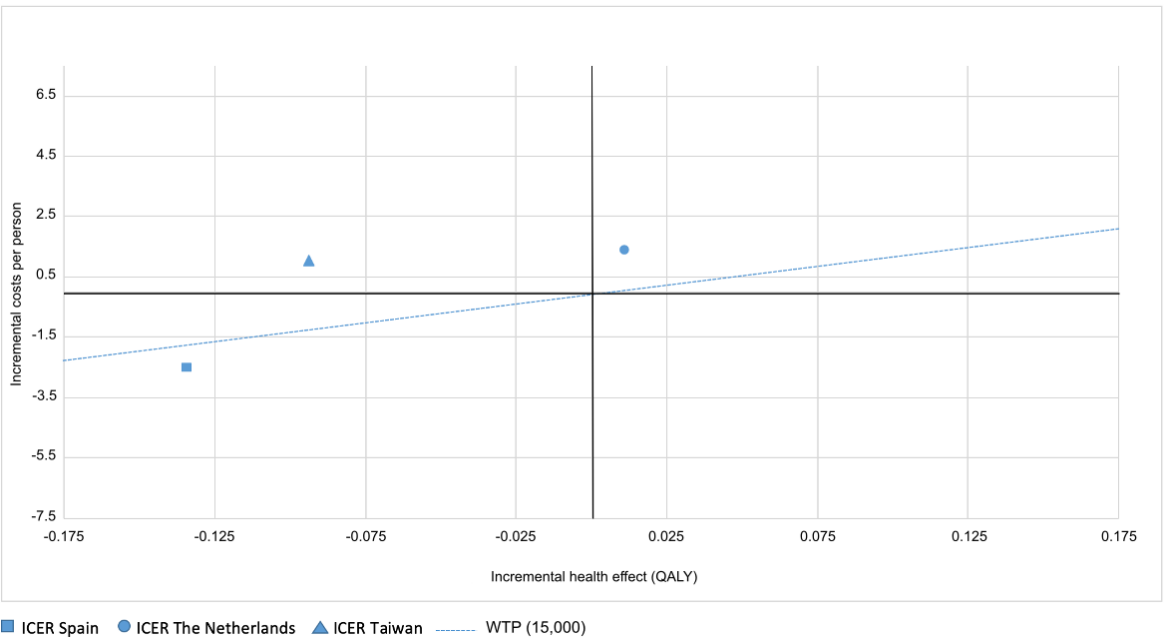
Cost-effectiveness plane for the Do CHANGE intervention in Spain, the Netherlands, and Taiwan. The dotted line shows the willingness-to-pay threshold of €15,000 per QALY. Do CHANGE: Do Cardiac Health: Advanced New Generation Ecosystem; ICER: incremental cost-effectiveness ratio; QALY: quality-adjusted life-year; WTP: willingness to pay.

After cleaning the data, 207 participants were included in the cost-effectiveness analysis (92 in the intervention group and 115 in the control group). The Do CHANGE 2 intervention was less costly in Spain (incremental cost was −€2514.90) and more costly in the Netherlands and Taiwan (incremental costs were €1373.59 and €1062.54, respectively). Figure 4 shows the cost-effectiveness plane for the three countries. The cost-effectiveness plane plots the incremental cost of the intervention on the y-axis and the incremental health outcome (measured in QALYs) on the x-axis. The diagonal line represents the WTP per additional QALY gained, which is the maximum amount that the society is willing to give in exchange for a better quality of life. Different thresholds may also be selected. Depending on the location of the ICER point in this plane, one would be able to interpret whether an intervention is cost-effective. When the ICER point is within the lower-right quadrant, it means the intervention is accepted (it is more effective and cheaper), and when it is within the upper-left quadrant, it means that the intervention is not accepted (it is less effective and more expensive). If the ICER point lies in the other two quadrants, then the intervention may or may not be accepted depending on the ICER and WTP threshold values.

Compared with usual care, the effectiveness of the Do CHANGE 2 program in terms of QALY gains was slightly higher in the Netherlands (incremental effect of 0.011) and lower in Spain and Taiwan (incremental effects of −0.134 and −0.094, respectively). Even though the Do CHANGE program was more effective than usual care in the Netherlands, the relative costs for gained utility (€124,489.27 per QALY) were too high to accept this intervention. Taken together, the Do CHANGE intervention would only be accepted in Spain, where it would help save €18,769.05 per QALY.

We also calculated the incremental cost and health-related quality of life for every age-gender combination in the specified target population. The data are presented in Multimedia Appendix 4.

## Discussion

### Principal Findings

In this cost-effectiveness analysis of an ICT-based intervention to change the health behavior of patients with CVD (the Do CHANGE program) assessed in a multicenter RCT, we found that the ICER strongly varied depending on the country where the intervention was applied. The Do CHANGE 2 program was slightly more effective than usual care in the Netherlands only, albeit at an incremental cost too high to accept the intervention at the selected WTP threshold (€15,000 per QALY). The same intervention was less effective but less costly than usual care in Spain. In Taiwan, the intervention resulted in the dominated option (less effective and more expensive). Therefore, implementation of the Do CHANGE 2 intervention is only recommended in Spain, where it could allow saving financial costs taking into account the costs and effects of the intervention. We further tested the results with higher WTP thresholds (ie, €30,000 per QALY), with results remaining in the same line.

### Contextualization With Previous Work

There is a large body of evidence showing that ICT solutions, including mobile-based telemonitoring, improve the quality and outcomes of care in patients with CVD [46]. Unfortunately, the cost-effectiveness assessment is often disregarded, and many studies reporting cost information do not meet a quality standard high enough to determine the cost-effectiveness or cost-utility of the intervention [46,47].

Regardless of the quality in reporting of individual studies, evidence on the cost-effectiveness of ICT-based lifestyle interventions is rather controversial, and many authors have acknowledged difficulties in drawing strong conclusions in this regard [48-50]. Overall, cost-effectiveness evaluations of secondary and tertiary prevention strategies for patients with CVD are challenged by the multiple factors influencing the outcomes and costs, such as baseline cardiovascular risk, the cost of drugs or other interventions, reimbursement procedures, and implementation of preventive strategies [1]. In the case of telemedicine approaches, it has been recognized that cost-effectiveness depends largely on local aspects of the individual service (and care as usual) being evaluated, and a service may be highly cost-effective in one context but highly ineffective when transferred to another context [47]. This was the case in our analysis, which yielded controversial results regarding the cost-effectiveness of the intervention in the different countries involved. Importantly, the success of an ICT-based lifestyle intervention strongly depends on the willingness of individuals to adopt the intervention, which is likely to be associated with cultural constraints and, therefore, to be country specific.

These differences were particularly pervasive between the Netherlands and Taiwan, where the cost-effectiveness planes showed an almost opposite profile, although the same intervention was implemented. We associate this situation with the majority of patients recruited in Taiwan having hypertension as the primary diagnosis and medical consultations involving health care professionals (ie, physicians) in the Netherlands being too expensive for them to devote time to a prevention intervention that could perhaps be conducted by nurses. This finding supports the need to evaluate the cost-effectiveness of these types of interventions within each context in order to provide the various stakeholders with evidence to understand the financial consequences of scaling up ICT solutions for health care.

### Limitations

The main limitation of our trial was the sample size, which was constrained by budget restrictions. The discrepancy between the target and the actual sample size was mainly due to technical difficulties in recruiting participants, who had to enroll in the trial for a minimum duration of 6 months. The low sample size might have constrained the representativeness of the results and made them more sensitive to biases associated with patients with extreme behaviors (ie, outliers). Actually, the extremely low number of participants within, for instance, the states “baseline” and “progressive disease stage 1,” might explain the unrealistic utilities of these patients before the intervention (eg, 0.962 and 1, respectively, for the control group), which were considerably higher than the average reported in larger RCTs involving HF (utility 0.84) [51]. Another example associated with this limitation is the relevant differences for patients allocated to the intervention group in Spain, who were younger and had higher education.

Second, an acknowledgment must be made regarding the limitation associated with the heterogeneous characteristics of the study sample for the primary diagnosis and cultural setting (ie, Spain, the Netherlands, and Taiwan), which may have contributed to the heterogeneous results across countries.

Unlike other cost-effectiveness analyses, we did not consider the contribution of medication to the health care costs. Although this might have increased the accuracy of the absolute costs, from a clinical point of view, it is unrealistic that the prescribed medicines would change throughout a 3-month time lapse. Since our main interest was assessing the change in costs rather than describing the actual values, we considered that it was more appropriate to exclude this concept from the cost-effectiveness analysis.

Finally, although RCTs are considered the gold standard for assessing cost-effectiveness, some authors criticize that they might miss information regarding how the intervention fits into routine practice [15].

### Conclusions

Our results suggest that the Do CHANGE 2 environment may help reduce health care costs associated with the management of patients with CVD in certain settings. However, changing health behavior and assessing the impact of this change on health care and societal costs remain big challenges. In line with previous research in this field, our assessment does not allow drawing strong conclusions in this regard. Irrespective of the specific cost-effectiveness of the Do CHANGE 2 program, our results highlight the high heterogeneity that ICT-based interventions might show depending on the country where they are implemented and stress the need for assessing each intervention in all areas before scaling up implementation.

## Appendix

Multimedia Appendix 1 Do CHANGE leaflet.

Multimedia Appendix 2 Clinical characteristics of the study sample, medication, and psychological symptoms.

Multimedia Appendix 3 Health care and societal costs.

Multimedia Appendix 4 Incremental costs and effects (age and gender specific) per pilot site.

## Abbreviations

BP: blood pressure
CVD: cardiovascular disease
DBP: diastolic blood pressure
DSD: Do Something Different
Do CHANGE: Do Cardiac Health: Advanced New Generation Ecosystem
EQ-5D: EuroQol five dimensions
HF: heart failure
ICER: incremental cost-effectiveness ratio
ICT: information and communication technology
MAFEIP: Monitoring and Assessment Framework for the European Innovation Partnership on Active and Healthy Ageing
QALY: quality-adjusted life-year
RCT: randomized controlled trial
SBP: systolic blood pressure
TAU: treatment as usual
WTP: willingness to pay
