# Supplementary material for: Changing the Health Behavior of Patients With Cardiovascular Disease Through an Electronic Health Intervention in Three Different Countries: Cost-Effectiveness Study in the Do Cardiac Health: Advanced New Generation Ecosystem (Do CHANGE) 2 Randomized Controlled Trial
Source: J Med Internet Res. 2020 Jul 28;22(7):e17351. doi: 10.2196/17351 (PMC7420510; doi:10.2196/17351)
Supplement: Multimedia Appendix 1 [file jmir_v22i7e17351_app1.pdf]

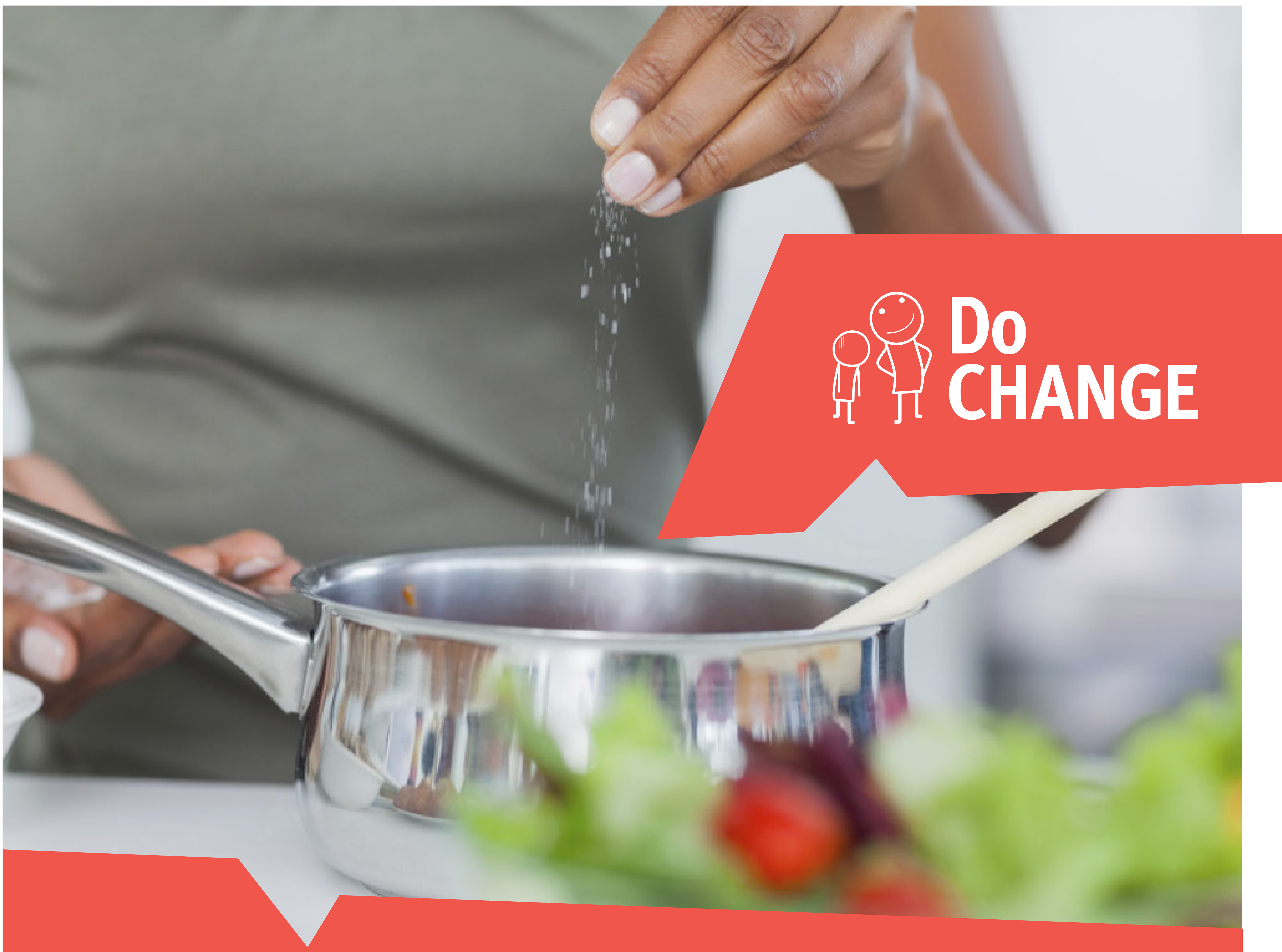

Do Cardiac Health: Advanced New Generation Ecosystem

February 2015- April 2018

- Self-management programme for people with cardiac diseases to change their habits and learn to enjoy a healthier lifestyle;
- Pesonalisation based on the person's habits, personality and real-time information from different sensors and existing medical devices;
- Person controlled data storage and access on nutrition, activity, hydration, health, behaviour and habits.

Do CHANGE for a better and healthier lifestyle!

Co-funded by the Horizon 2020
Framework Programme of the European Union
(4,7 million)

Co-funded by the department
of industrial technology Taiwan
(1 million)
