# Supplementary material for: Changing the Health Behavior of Patients With Cardiovascular Disease Through an Electronic Health Intervention in Three Different Countries: Cost-Effectiveness Study in the Do Cardiac Health: Advanced New Generation Ecosystem (Do CHANGE) 2 Randomized Controlled Trial
Source: J Med Internet Res. 2020 Jul 28;22(7):e17351. doi: 10.2196/17351 (PMC7420510; doi:10.2196/17351)
Supplement: Multimedia Appendix 2 [file jmir_v22i7e17351_app2.pdf]

		Spain	The Netherlands	Taiwan	Total
**Primary diagnoses**					
Heart Failure *n (%)*	Do CHANGE (*intervention condition*)	7 (18.4)	9 (23.7)	4 (9.1)	20 (16.7)
	Care-as-usual (*control condition*)	9 (24.3)	8 (21.6)	2 (4.5)	19 (16.1)
	Total	16 (21.3)	17 (22.7)	6 (6.8)	39 (16.4)
Hypertension *n (%)*	Do CHANGE (*intervention condition*)	24 (63.2)	11 (28.9)	27 (61.4)	62 (51.7)
	Care-as-usual (*control condition*)	22 (59.5)	10 (27.0)	31 (70.5)	63 (53.4)
	Total	46 (61.3)	21 (28.0)	58 (65.9)	125 (52.5)
Cardiovascular Artery Disease *n (%)*	Do CHANGE (*intervention condition*)	7 (18.4)	18 (47.4)	13 (29.5)	38 (31.7)
	Care-as-usual (*control condition*)	6 (16.2)	19 (51.4)	11 (25.0)	36 (30.5)
	Total	13 (17.3)	37 (49.3)	24 (27.3)	74 (31.1)
**Other clinical characteristics**					
Diabetes Mellitus *n (%)*	Do CHANGE (*intervention condition*)	9 (23.7)	9 (23.7)	4 (9.1)	22 (18.3)
	Care-as-usual (*control condition*)	6 (16.2)	9 (24.3)	1 (2.3)	16 (13.6)
	Total	15 (20.0)	18 (24.0)	5 (5.7)	38 (16.0)
SBP (mmHg) *mean (SD)*	Do CHANGE (*intervention condition*)	133.24 (18.92)	143.50 (27.44)	128.68 (18.68)	134.88 (22.57)
	Care-as-usual (*control condition*)	135.78 (13.92)	143.81 (22.13)	133.27 (12.84)	137.36 (17.06)
	Total	134.49 (16.58)	143.65 (24.80)	131.07 (16.09)	136.11 (20.03)
DBP (mmHg) *mean (SD)*	Do CHANGE (*intervention condition*)	77.87 (11.35)	83.63 (15.58)	76.82 (13.76)	79.31 (13.89)
	Care-as-usual (*control condition*)	77.78 (9.67)	81.14 (12.23)	82.57 (9.61)	80.62 (10.62)
	Total	77.83 (10.49)	82.40 (13.00)	79.69 (12.15)	79.96 (12.37)

Heart Rate (rest; beats/min) *mean (SD)*	Do CHANGE (*intervention condition*)	72.03 (10.17)	68.34 (14.33)	68.48 (12.65)	69.56 (12.52)
	Care-as-usual (*control condition*)	71.08 (10.05)	67.14 (12.71)	71.77 (11.00)	70.10 (11.38)
	Total	71.56 (10.05)	67.75 (13.48)	70.13 (11.90)	69.83 (11.94)
CCI score *mean (SD)*	Do CHANGE (*intervention condition*)	0.79 (0.88)	1.32 (0.99)	0.66 (1.10)	0.91 (1.03)
	Care-as-usual (*control condition*)	1.03 (0.99)	1.41 (0.83)	0.32 (0.52)	0.88 (0.91)
	Total	0.91 (0.93))	1.36 (0.91)	0.49 (0.87)	0.89 (0.97)
BMI (kg/m^2) *mean (SD)*	Do CHANGE (*intervention condition*)	29.0 (5.4)	29.3 (4.3)	25.4 (4.2)	27.8 (5.0)
	Care-as-usual (*control condition*)	29.4 (6.0)	29.1 (5.0)	26.1 (2.7)	28.1 (4.9)
	Total	29.2 (5.7)	29.2 (4.6)	25.8 (3.6)	27.9 (4.9)
**Medication** *n (%)*					
Antiplatelet	Do CHANGE (*intervention condition*)	11 (28.9)	34 (89.5)	15 (34.1)	60 (50.0)
	Care-as-usual (*control condition*)	17 (45.9)	25 (67.6)	11 (25.0)	53 (44.9)
	Total	28 (37.3)	59 (78.7)	26 (29.5)	113 (47.5)
Statins	Do CHANGE (*intervention condition*)	19 (50.0)	28 (73.7)	28 (63.6)	75 (62.5)
	Care-as-usual (*control condition*)	22 (59.5)	24 (64.9)	21 (47.7)	67 (56.8)
	Total	41 (54.7)	52 (69.3)	49 (55.7)	142 (59.7)
Beta-blockers	Do CHANGE (*intervention condition*)	15 (39.5)	28 (73.7)	23 (52.3)	66 (55.0)
	Care-as-usual (*control condition*)	18 (48.6)	23 (62.2)	14 (31.8)	55 (46.6)
	Total	33 (44.0)	51 (68.0)	37 (42.0)	121 (50.8)
ACE-inhibitors	Do CHANGE (*intervention condition*)	14 (36.8)	19 (50.0)	11 (25.0)	44 (36.7)
	Care-as-usual (*control condition*)	8 (21.6)	13 (35.1)	19 (43.2)	40 (33.9)
	Total	22 (29.3)	32 (42.7)	30 (34.1)	84 (35.3)

ARB	Do CHANGE (*intervention condition*)	12 (31.3)	11 (28.9)	12 (27.3)	35 (29.2)
	Care-as-usual (*control condition*)	17 (45.9)	10 (27.0)	12 (27.3)	39 (33.1)
	Total	29 (38.7)	21 (28.0)	24 (27.3)	74 (31.1)
Calcium antagonist	Do CHANGE (*intervention condition*)	6 (15.8)	9 (23.7)	15 (34.1)	30 (25.0)
	Care-as-usual (*control condition*)	11 (29.7)	8 (21.6)	15 (34.1)	34 (28.8)
	Total	17 (22.7)	17 (22.7)	30 (34.1)	64 (26.9)
Psychotropic medication	Do CHANGE (*intervention condition*)	8 (21.1)	4 (10.5)	7 (15.9)	19 (15.8)
	Care-as-usual (*control condition*)	17 (45.9)	6 (16.2)	7 (15.9)	30 (25.4)
	Total	25 (33.3)	10 (13.3)	14 (15.0)	49 (20.6)
**Psychological symptoms**					
PHQ-9 *mean (SD)*	Do CHANGE (*intervention condition*)	3.9 (3.0)	3.8 (4.3)	5.6 (5.1)	4.5 (4.3)
	Care-as-usual (*control condition*)	4.4 (3.2)	4.0 (3.7)	2.9 (2.6)	3.7 (3.2)
	Total	4.2 (3.1)	3.9 (4.0)	4.3 (4.3)	4.1 (3.8)
GAD-7 *mean (SD)*	Do CHANGE (*intervention condition*)	4.3 (3.3)	2.8 (3.5)	3.7 (4.0)	3.6 (3.6)
	Care-as-usual (*control condition*)	4.3 (2.8)	3.1 (4.0)	3.6 (4.1)	6.7 (3.7)
	Total	4.3 (3.0)	3.0 (3.7)	3.6 (4.0)	3.6 (3.7)
Type D personality *n (%)*	Do CHANGE (*intervention condition*)	4 (10.5)	6 (15.8)	10 (22.7)	20 (16.7)
	Care-as-usual (*control condition*)	3 (8.1)	8 (21.6)	7 (15.9)	18 (15.3)
	Total	7 (9.3)	14 (18.7)	17 (19.3)	38 (16.0)
HPLP II *mean (SD)*	Do CHANGE (*intervention condition*)	136.2 (27.2)	128.0 (19.7)	142.1 (23.6)	135.7 (24.2)
	Care-as-usual (*control condition*)	143.5 (19.6)	128.7 (18.4)	141.5 (20.7)	138.1 (20.5)

	Total	139.8 (23.9)	128.3 (18.9)	141.8 (22.1)	136.9 (22.4)
QoL<br>*mean (SD)*	Do CHANGE (*intervention condition*)	101.1 (9.9)	99.3 (15.9)	93.5 (11.7)	97.7 (13.0)
	Care-as-usual (*control condition*)	96.8 (12.7)	99.7 (13.3)	92.8 (9.4)	96.2 (12.1)
	Total	99.0 (11.5)	99.5 (14.6)	93.1 (10.6)	97.0 (12.5)

ACE: Angiotensin-Converting-Enzyme; **ARB:** Angiotensin II Receptor Blocker; **BMI**: Body Mass Index; **CAD**: Coronary Artery Disease; **CCI:** Charlson Comorbidity Index; **DBP:** Diastolic Blood Pressure; **GAD-7**: General Anxiety Disorder-7; **HCU**: healthcare utilization; **HF**: Heart failure; **HPLP II**: Health Promoting Lifestyle Profile II questionnaire; **HT**: Hypertension; **PHQ-9**: Patient Health Questionnaire - 9; **SBP**: Systolic Blood Pressure; **SD**: Standard Deviation; **QoL:** Quality of Life.
