# Supplementary material for: Changing the Health Behavior of Patients With Cardiovascular Disease Through an Electronic Health Intervention in Three Different Countries: Cost-Effectiveness Study in the Do Cardiac Health: Advanced New Generation Ecosystem (Do CHANGE) 2 Randomized Controlled Trial
Source: J Med Internet Res. 2020 Jul 28;22(7):e17351. doi: 10.2196/17351 (PMC7420510; doi:10.2196/17351)
Supplement: Multimedia Appendix 4 [file jmir_v22i7e17351_app4.pdf]

Incremental costs and effects (age and gender specific)

Incremental cost and health-related quality of life are shown here for every age-gender combination in the specified target population. On the one hand, incremental costs refer to the difference between the cost that a person from a specific age and gender would have if he/she received the intervention minus the cost that would have if he/she followed current care. On the other hand, the incremental effects values show how much quality of life (utility) is gained when the intervention is used instead of the standard care. These are also shown per age-gender group. Both the discounted and the undiscounted incremental outcomes are presented. This information is also provided in the tables below per each pilot site participating in the Do CHANGE 2 project (Spain, the Netherlands and Taiwan).

Spain

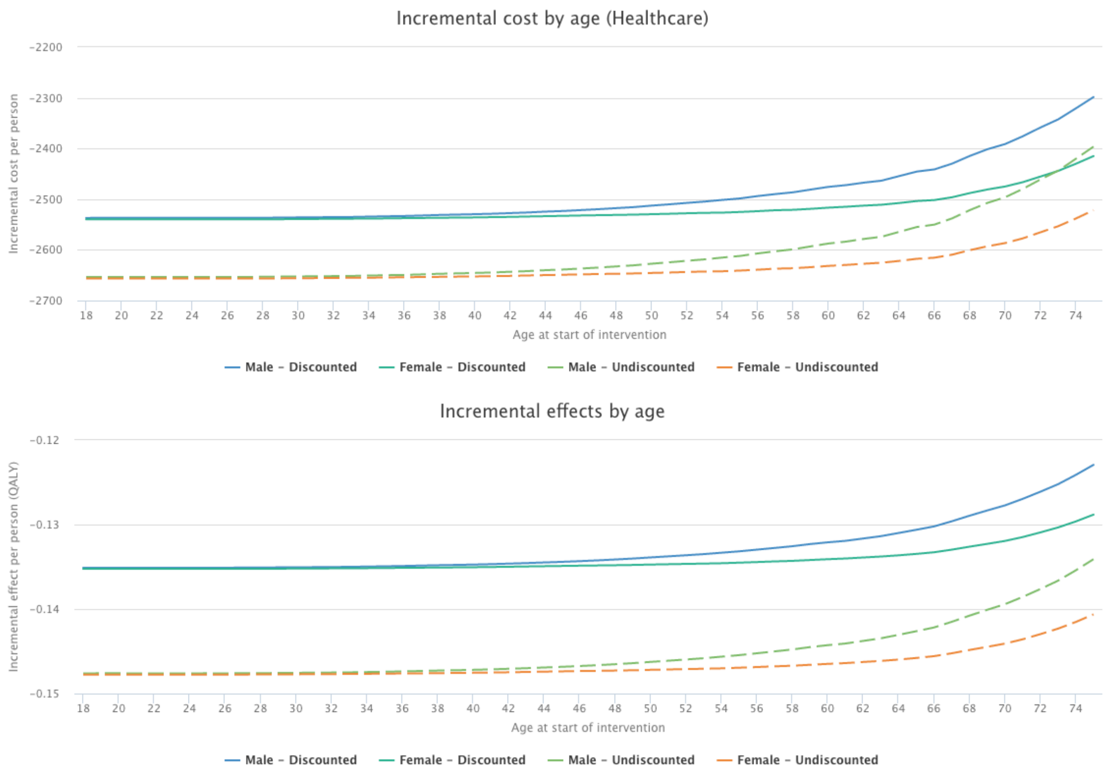

Figure 1: Incremental cost and effects by age for Spain.

Table 1: Incremental costs and effects for Spain (Male)

Age	Incremental cost (Healthcare)	Incremental effects	Undiscounted incremental cost (Healthcare)	Undiscounted incremental effects
18	-2537.03	-0.135	-2653.57	-0.148
19	-2536.78	-0.135	-2653.31	-0.148
20	-2536.78	-0.135	-2653.31	-0.148
21	-2536.82	-0.135	-2653.35	-0.148
22	-2536.88	-0.135	-2653.42	-0.148
23	-2536.95	-0.135	-2653.49	-0.148
24	-2536.91	-0.135	-2653.44	-0.148
25	-2536.76	-0.135	-2653.29	-0.148
26	-2536.87	-0.135	-2653.40	-0.148
27	-2536.77	-0.135	-2653.29	-0.148
28	-2536.72	-0.135	-2653.23	-0.148
29	-2536.29	-0.135	-2652.77	-0.148
30	-2535.95	-0.135	-2652.41	-0.148
31	-2535.62	-0.135	-2652.05	-0.148
32	-2535.35	-0.135	-2651.76	-0.148
33	-2535.11	-0.135	-2651.49	-0.147
34	-2534.38	-0.135	-2650.71	-0.147
35	-2533.68	-0.135	-2649.96	-0.147
36	-2533.17	-0.135	-2649.39	-0.147
37	-2532.18	-0.135	-2648.33	-0.147
38	-2531.09	-0.135	-2647.16	-0.147
39	-2530.33	-0.135	-2646.34	-0.147
40	-2529.57	-0.135	-2645.52	-0.147
41	-2528.69	-0.135	-2644.56	-0.147
42	-2527.42	-0.135	-2643.20	-0.147
43	-2526.18	-0.135	-2641.86	-0.147
44	-2524.49	-0.134	-2640.04	-0.147
45	-2523.26	-0.134	-2638.70	-0.147
46	-2521.48	-0.134	-2636.78	-0.147
47	-2519.70	-0.134	-2634.87	-0.147
48	-2517.61	-0.134	-2632.61	-0.147
49	-2515.59	-0.134	-2630.42	-0.146
50	-2512.65	-0.134	-2627.25	-0.146
51	-2510.10	-0.134	-2624.50	-0.146
52	-2507.29	-0.134	-2621.48	-0.146

Age	Incremental cost (Healthcare)	Incremental effects	Undiscounted incremental cost (Healthcare)	Undiscounted incremental effects
53	-2504.67	-0.133	-2618.65	-0.146
54	-2501.44	-0.133	-2615.16	-0.146
55	-2498.51	-0.133	-2611.98	-0.145
56	-2493.86	-0.133	-2606.99	-0.145
57	-2489.93	-0.133	-2602.75	-0.145
58	-2486.44	-0.133	-2598.96	-0.145
59	-2481.00	-0.132	-2593.11	-0.145
60	-2475.73	-0.132	-2587.46	-0.144
61	-2472.25	-0.132	-2583.71	-0.144
62	-2467.38	-0.132	-2578.43	-0.144
63	-2463.50	-0.131	-2574.18	-0.143
64	-2454.47	-0.131	-2564.49	-0.143
65	-2445.52	-0.131	-2554.91	-0.143
66	-2441.23	-0.130	-2550.13	-0.142
67	-2429.84	-0.130	-2537.82	-0.142
68	-2414.68	-0.129	-2521.56	-0.141
69	-2401.42	-0.128	-2507.30	-0.140
70	-2391.23	-0.128	-2496.25	-0.139
71	-2375.90	-0.127	-2479.72	-0.139
72	-2358.58	-0.126	-2461.09	-0.138
73	-2342.60	-0.125	-2443.80	-0.137
74	-2320.88	-0.124	-2420.42	-0.135
75	-2298.55	-0.123	-2396.32	-0.134

Table 2: Incremental costs and effects for Spain (Female)

Age	Incremental cost (Healthcare)	Incremental effects	Undiscounted incremental cost (Healthcare)	Undiscounted incremental effects
18	-2539.76	-0.135	-2656.52	-0.148
19	-2539.80	-0.135	-2656.56	-0.148
20	-2539.78	-0.135	-2656.54	-0.148
21	-2539.77	-0.135	-2656.53	-0.148
22	-2539.78	-0.135	-2656.54	-0.148
23	-2539.75	-0.135	-2656.51	-0.148
24	-2539.70	-0.135	-2656.45	-0.148

Age	Incremental cost (Healthcare)	Incremental effects	Undiscounted incremental cost (Healthcare)	Undiscounted incremental effects
25	-2539.72	-0.135	-2656.47	-0.148
26	-2539.60	-0.135	-2656.35	-0.148
27	-2539.61	-0.135	-2656.36	-0.148
28	-2539.59	-0.135	-2656.33	-0.148
29	-2539.44	-0.135	-2656.17	-0.148
30	-2539.23	-0.135	-2655.94	-0.148
31	-2538.95	-0.135	-2655.64	-0.148
32	-2538.67	-0.135	-2655.34	-0.148
33	-2538.45	-0.135	-2655.10	-0.148
34	-2538.22	-0.135	-2654.85	-0.148
35	-2537.87	-0.135	-2654.47	-0.148
36	-2537.38	-0.135	-2653.95	-0.148
37	-2537.03	-0.135	-2653.57	-0.148
38	-2536.72	-0.135	-2653.23	-0.148
39	-2536.27	-0.135	-2652.75	-0.148
40	-2535.96	-0.135	-2652.41	-0.148
41	-2535.36	-0.135	-2651.77	-0.148
42	-2534.94	-0.135	-2651.30	-0.147
43	-2534.14	-0.135	-2650.45	-0.147
44	-2533.47	-0.135	-2649.73	-0.147
45	-2532.86	-0.135	-2649.06	-0.147
46	-2532.11	-0.135	-2648.26	-0.147
47	-2531.54	-0.135	-2647.65	-0.147
48	-2531.06	-0.135	-2647.13	-0.147
49	-2530.43	-0.135	-2646.44	-0.147
50	-2529.58	-0.135	-2645.53	-0.147
51	-2528.75	-0.135	-2644.63	-0.147
52	-2527.72	-0.135	-2643.53	-0.147
53	-2526.94	-0.135	-2642.69	-0.147
54	-2526.48	-0.135	-2642.18	-0.147
55	-2525.20	-0.135	-2640.80	-0.147
56	-2523.61	-0.134	-2639.08	-0.147
57	-2521.75	-0.134	-2637.09	-0.147
58	-2520.94	-0.134	-2636.20	-0.147
59	-2519.00	-0.134	-2634.10	-0.147
60	-2516.68	-0.134	-2631.61	-0.146

Age	Incremental cost (Healthcare)	Incremental effects	Undiscounted incremental cost (Healthcare)	Undiscounted incremental effects
61	-2514.88	-0.134	-2629.67	-0.146
62	-2512.68	-0.134	-2627.29	-0.146
63	-2510.92	-0.134	-2625.37	-0.146
64	-2507.56	-0.134	-2621.76	-0.146
65	-2503.65	-0.133	-2617.57	-0.146
66	-2501.68	-0.133	-2615.37	-0.146
67	-2496.20	-0.133	-2609.43	-0.145
68	-2487.95	-0.133	-2600.57	-0.145
69	-2480.85	-0.132	-2592.93	-0.144
70	-2474.96	-0.132	-2586.54	-0.144
71	-2466.42	-0.131	-2577.30	-0.144
72	-2455.28	-0.131	-2565.29	-0.143
73	-2444.20	-0.130	-2553.32	-0.142
74	-2430.16	-0.130	-2538.17	-0.142
75	-2414.96	-0.129	-2521.75	-0.141

The Netherlands

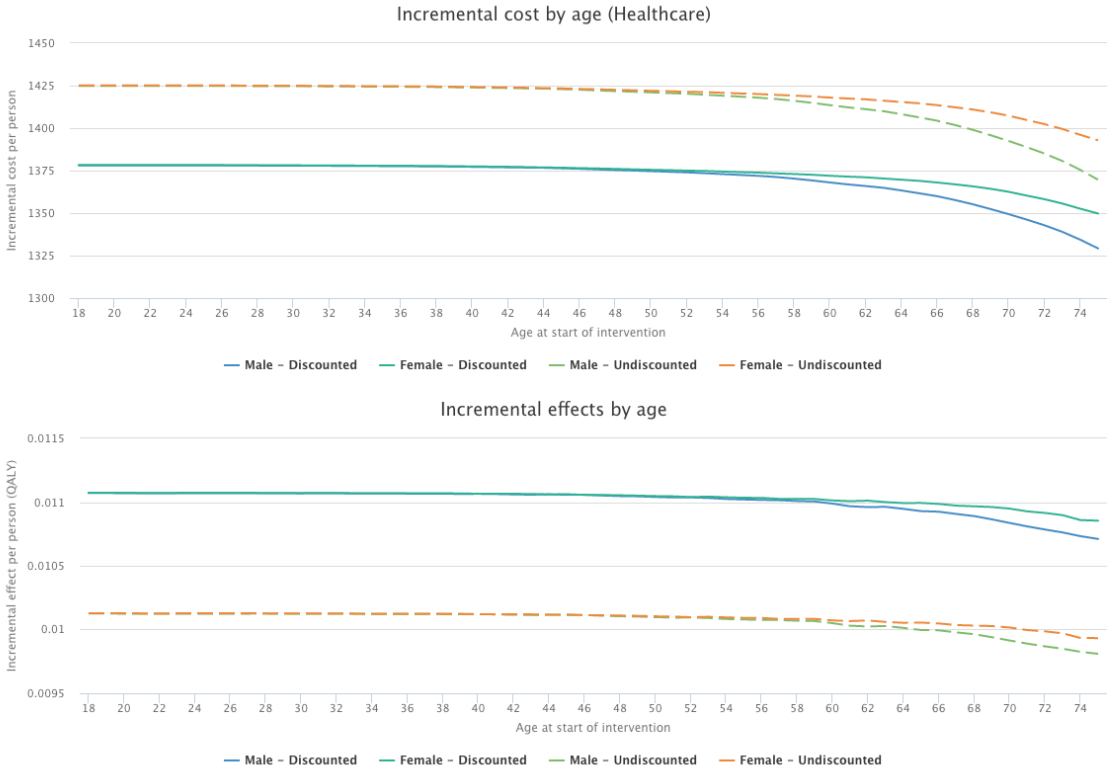

Figure 2: Incremental cost and effects by age for the Netherlands.

Table 3: Incremental costs and effects for the Netherlands (Male)

Age	Incremental cost (Healthcare)	Incremental effects	Undiscounted incremental cost (Healthcare)	Undiscounted incremental effects
18	1378.10	0.011	1424.91	0.010
19	1378.08	0.011	1424.88	0.010
20	1378.03	0.011	1424.83	0.010
21	1378.04	0.011	1424.84	0.010
22	1378.04	0.011	1424.83	0.010
23	1378.02	0.011	1424.82	0.010
24	1378.04	0.011	1424.84	0.010
25	1378.03	0.011	1424.83	0.010
26	1378.02	0.011	1424.82	0.010
27	1378.00	0.011	1424.80	0.010
28	1377.98	0.011	1424.77	0.010
29	1377.94	0.011	1424.73	0.010
30	1377.91	0.011	1424.70	0.010
31	1377.90	0.011	1424.68	0.010
32	1377.84	0.011	1424.61	0.010
33	1377.79	0.011	1424.56	0.010
34	1377.73	0.011	1424.49	0.010
35	1377.68	0.011	1424.43	0.010
36	1377.62	0.011	1424.36	0.010
37	1377.55	0.011	1424.28	0.010
38	1377.47	0.011	1424.19	0.010
39	1377.35	0.011	1424.06	0.010
40	1377.24	0.011	1423.94	0.010
41	1377.10	0.011	1423.78	0.010
42	1376.93	0.011	1423.59	0.010
43	1376.80	0.011	1423.44	0.010
44	1376.69	0.011	1423.31	0.010
45	1376.37	0.011	1422.95	0.010
46	1376.09	0.011	1422.63	0.010
47	1375.70	0.011	1422.19	0.010
48	1375.34	0.011	1421.79	0.010
49	1375.04	0.011	1421.44	0.010
50	1374.69	0.011	1421.05	0.010
51	1374.33	0.011	1420.64	0.010
52	1373.98	0.011	1420.24	0.010

Age	Incremental cost (Healthcare)	Incremental effects	Undiscounted incremental cost (Healthcare)	Undiscounted incremental effects
53	1373.49	0.011	1419.69	0.010
54	1372.96	0.011	1419.10	0.010
55	1372.47	0.011	1418.55	0.010
56	1371.93	0.011	1417.93	0.010
57	1371.25	0.011	1417.16	0.010
58	1370.31	0.011	1416.09	0.010
59	1369.25	0.011	1414.89	0.010
60	1368.04	0.011	1413.53	0.010
61	1366.89	0.011	1412.25	0.010
62	1365.92	0.011	1411.14	0.010
63	1364.89	0.011	1409.97	0.010
64	1363.33	0.011	1408.21	0.010
65	1361.66	0.011	1406.32	0.010
66	1359.95	0.011	1404.38	0.010
67	1357.69	0.011	1401.82	0.010
68	1355.19	0.011	1399.00	0.010
69	1352.35	0.011	1395.80	0.010
70	1349.38	0.011	1392.45	0.010
71	1346.21	0.011	1388.87	0.010
72	1342.86	0.011	1385.08	0.010
73	1338.99	0.011	1380.70	0.010
74	1334.39	0.011	1375.50	0.010
75	1329.22	0.011	1369.66	0.010

Table 4: Incremental costs and effects for the Netherlands (Female)

Age	Incremental cost (Healthcare)	Incremental effects	Undiscounted incremental cost (Healthcare)	Undiscounted incremental effects
18	1378.35	0.011	1425.19	0.010
19	1378.33	0.011	1425.16	0.010
20	1378.30	0.011	1425.14	0.010
21	1378.29	0.011	1425.12	0.010
22	1378.30	0.011	1425.14	0.010
23	1378.33	0.011	1425.16	0.010
24	1378.34	0.011	1425.18	0.010
25	1378.30	0.011	1425.13	0.010

Age	Incremental cost (Healthcare)	Incremental effects	Undiscounted incremental cost (Healthcare)	Undiscounted incremental effects
26	1378.27	0.011	1425.10	0.010
27	1378.24	0.011	1425.07	0.010
28	1378.19	0.011	1425.01	0.010
29	1378.18	0.011	1425.00	0.010
30	1378.13	0.011	1424.95	0.010
31	1378.11	0.011	1424.92	0.010
32	1378.07	0.011	1424.87	0.010
33	1378.00	0.011	1424.79	0.010
34	1377.96	0.011	1424.75	0.010
35	1377.92	0.011	1424.70	0.010
36	1377.88	0.011	1424.66	0.010
37	1377.82	0.011	1424.59	0.010
38	1377.73	0.011	1424.49	0.010
39	1377.62	0.011	1424.37	0.010
40	1377.50	0.011	1424.22	0.010
41	1377.38	0.011	1424.09	0.010
42	1377.27	0.011	1423.97	0.010
43	1377.09	0.011	1423.76	0.010
44	1376.88	0.011	1423.53	0.010
45	1376.75	0.011	1423.38	0.010
46	1376.47	0.011	1423.07	0.010
47	1376.28	0.011	1422.84	0.010
48	1376.03	0.011	1422.57	0.010
49	1375.75	0.011	1422.25	0.010
50	1375.51	0.011	1421.98	0.010
51	1375.29	0.011	1421.73	0.010
52	1375.00	0.011	1421.40	0.010
53	1374.84	0.011	1421.22	0.010
54	1374.46	0.011	1420.79	0.010
55	1374.11	0.011	1420.40	0.010
56	1373.82	0.011	1420.07	0.010
57	1373.38	0.011	1419.57	0.010
58	1373.01	0.011	1419.15	0.010
59	1372.57	0.011	1418.66	0.010
60	1371.98	0.011	1417.99	0.010
61	1371.48	0.011	1417.43	0.010

Age	Incremental cost (Healthcare)	Incremental effects	Undiscounted incremental cost (Healthcare)	Undiscounted incremental effects
62	1371.08	0.011	1416.97	0.010
63	1370.35	0.011	1416.15	0.010
64	1369.67	0.011	1415.37	0.010
65	1368.99	0.011	1414.60	0.010
66	1368.02	0.011	1413.51	0.010
67	1366.92	0.011	1412.27	0.010
68	1365.74	0.011	1410.93	0.010
69	1364.29	0.011	1409.29	0.010
70	1362.54	0.011	1407.31	0.010
71	1360.34	0.011	1404.82	0.010
72	1358.18	0.011	1402.38	0.010
73	1355.64	0.011	1399.51	0.010
74	1352.60	0.011	1396.08	0.010
75	1349.75	0.011	1392.85	0.010

Taiwan

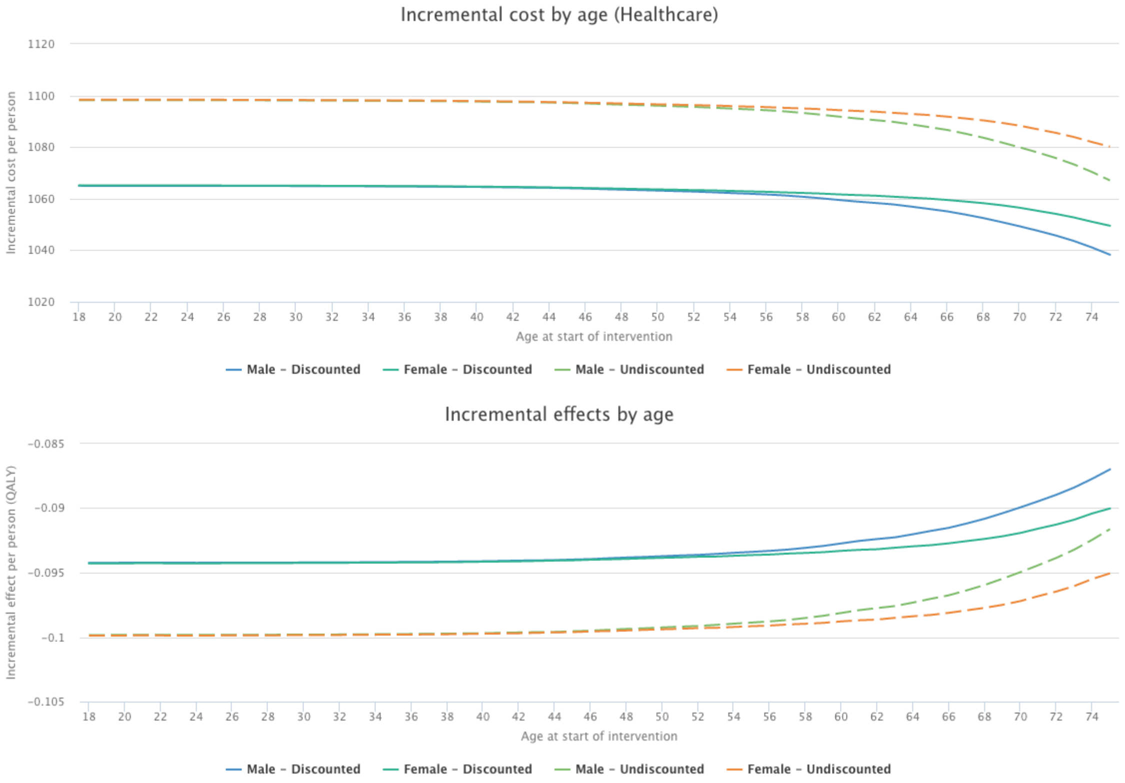

Figure 3: Incremental cost and effects by age for Taiwan.

Table 5: Incremental costs and effects for Taiwan (Male)

Age	Incremental cost (Healthcare)	Incremental effects	Undiscounted incremental cost (Healthcare)	Undiscounted incremental effects
18	1065.01	-0.094	1098.23	-0.100
19	1065.00	-0.094	1098.22	-0.100
20	1064.97	-0.094	1098.19	-0.100
21	1064.98	-0.094	1098.19	-0.100
22	1064.98	-0.094	1098.19	-0.100
23	1064.97	-0.094	1098.18	-0.100
24	1064.98	-0.094	1098.20	-0.100
25	1064.97	-0.094	1098.19	-0.100
26	1064.97	-0.094	1098.18	-0.100
27	1064.96	-0.094	1098.17	-0.100
28	1064.95	-0.094	1098.16	-0.100
29	1064.92	-0.094	1098.13	-0.100
30	1064.91	-0.094	1098.11	-0.100
31	1064.90	-0.094	1098.11	-0.100
32	1064.87	-0.094	1098.07	-0.100
33	1064.84	-0.094	1098.03	-0.100
34	1064.81	-0.094	1098.00	-0.100
35	1064.78	-0.094	1097.96	-0.100
36	1064.75	-0.094	1097.92	-0.100
37	1064.71	-0.094	1097.88	-0.100
38	1064.66	-0.094	1097.83	-0.100
39	1064.60	-0.094	1097.75	-0.100
40	1064.54	-0.094	1097.69	-0.100
41	1064.46	-0.094	1097.60	-0.100
42	1064.37	-0.094	1097.49	-0.100
43	1064.30	-0.094	1097.41	-0.100
44	1064.24	-0.094	1097.33	-0.100
45	1064.06	-0.094	1097.13	-0.100
46	1063.91	-0.094	1096.95	-0.100
47	1063.70	-0.094	1096.70	-0.099
48	1063.50	-0.094	1096.48	-0.099
49	1063.34	-0.094	1096.28	-0.099
50	1063.14	-0.094	1096.06	-0.099
51	1062.95	-0.094	1095.83	-0.099
52	1062.76	-0.094	1095.61	-0.099

Age	Incremental cost (Healthcare)	Incremental effects	Undiscounted incremental cost (Healthcare)	Undiscounted incremental effects
53	1062.49	-0.094	1095.30	-0.099
54	1062.20	-0.093	1094.96	-0.099
55	1061.93	-0.093	1094.65	-0.099
56	1061.64	-0.093	1094.30	-0.099
57	1061.27	-0.093	1093.87	-0.099
58	1060.75	-0.093	1093.26	-0.099
59	1060.17	-0.093	1092.59	-0.098
60	1059.51	-0.093	1091.83	-0.098
61	1058.88	-0.093	1091.10	-0.098
62	1058.35	-0.092	1090.48	-0.098
63	1057.79	-0.092	1089.82	-0.098
64	1056.94	-0.092	1088.82	-0.097
65	1056.02	-0.092	1087.76	-0.097
66	1055.09	-0.092	1086.67	-0.097
67	1053.85	-0.091	1085.22	-0.096
68	1052.48	-0.091	1083.63	-0.096
69	1050.93	-0.090	1081.83	-0.095
70	1049.31	-0.090	1079.94	-0.095
71	1047.57	-0.089	1077.92	-0.094
72	1045.74	-0.089	1075.78	-0.094
73	1043.61	-0.088	1073.31	-0.093
74	1041.09	-0.088	1070.37	-0.092
75	1038.27	-0.087	1067.08	-0.092

Table 6: Incremental costs and effects for Taiwan (Female)

Age	Incremental cost (Healthcare)	Incremental effects	Undiscounted incremental cost (Healthcare)	Undiscounted incremental effects
18	1065.15	-0.094	1098.39	-0.100
19	1065.13	-0.094	1098.37	-0.100
20	1065.12	-0.094	1098.36	-0.100
21	1065.11	-0.094	1098.35	-0.100
22	1065.12	-0.094	1098.36	-0.100
23	1065.13	-0.094	1098.38	-0.100
24	1065.14	-0.094	1098.39	-0.100

Age	Incremental cost (Healthcare)	Incremental effects	Undiscounted incremental cost (Healthcare)	Undiscounted incremental effects
25	1065.12	-0.094	1098.36	-0.100
26	1065.11	-0.094	1098.34	-0.100
27	1065.09	-0.094	1098.32	-0.100
28	1065.06	-0.094	1098.29	-0.100
29	1065.06	-0.094	1098.29	-0.100
30	1065.03	-0.094	1098.25	-0.100
31	1065.02	-0.094	1098.24	-0.100
32	1064.99	-0.094	1098.21	-0.100
33	1064.95	-0.094	1098.17	-0.100
34	1064.93	-0.094	1098.14	-0.100
35	1064.91	-0.094	1098.12	-0.100
36	1064.89	-0.094	1098.09	-0.100
37	1064.86	-0.094	1098.05	-0.100
38	1064.81	-0.094	1098.00	-0.100
39	1064.75	-0.094	1097.93	-0.100
40	1064.68	-0.094	1097.85	-0.100
41	1064.62	-0.094	1097.77	-0.100
42	1064.56	-0.094	1097.70	-0.100
43	1064.46	-0.094	1097.59	-0.100
44	1064.34	-0.094	1097.45	-0.100
45	1064.27	-0.094	1097.37	-0.100
46	1064.12	-0.094	1097.19	-0.100
47	1064.01	-0.094	1097.07	-0.100
48	1063.88	-0.094	1096.92	-0.099
49	1063.73	-0.094	1096.74	-0.099
50	1063.59	-0.094	1096.58	-0.099
51	1063.47	-0.094	1096.44	-0.099
52	1063.31	-0.094	1096.26	-0.099
53	1063.23	-0.094	1096.16	-0.099
54	1063.02	-0.094	1095.92	-0.099
55	1062.83	-0.094	1095.69	-0.099
56	1062.67	-0.094	1095.51	-0.099
57	1062.43	-0.094	1095.23	-0.099
58	1062.22	-0.093	1094.99	-0.099
59	1061.99	-0.093	1094.71	-0.099
60	1061.66	-0.093	1094.34	-0.099

Age	Incremental cost (Healthcare)	Incremental effects	Undiscounted incremental cost (Healthcare)	Undiscounted incremental effects
61	1061.39	-0.093	1094.02	-0.099
62	1061.17	-0.093	1093.76	-0.099
63	1060.78	-0.093	1093.30	-0.099
64	1060.40	-0.093	1092.86	-0.098
65	1060.03	-0.093	1092.43	-0.098
66	1059.50	-0.093	1091.81	-0.098
67	1058.90	-0.093	1091.11	-0.098
68	1058.25	-0.092	1090.36	-0.098
69	1057.46	-0.092	1089.43	-0.097
70	1056.51	-0.092	1088.32	-0.097
71	1055.30	-0.092	1086.91	-0.097
72	1054.12	-0.091	1085.53	-0.096
73	1052.73	-0.091	1083.92	-0.096
74	1051.06	-0.090	1081.99	-0.095
75	1049.51	-0.090	1080.16	-0.095
